# Temporal and Contextual Consistency of Leadership in Homing Pigeon Flocks

**DOI:** 10.1371/journal.pone.0102771

**Published:** 2014-07-23

**Authors:** Carlos D. Santos, Stefanie Neupert, Hans-Peter Lipp, Martin Wikelski, Dina K. N. Dechmann

**Affiliations:** 1 Department of Migration and Immuno-ecology, Max Planck Institute for Ornithology, Am Obstberg 1, Radolfzell, Germany; 2 Departamento de Biologia, Centro de Ciências Biológicas e da Saúde, Universidade Federal do Maranhão, Campus do Bacanga, São Luís, Maranhão, Brazil; 3 Centro de Estudos do Ambiente e do Mar, Departamento de Biologia, Universidade de Aveiro, Campus Universitário de Santiago, Aveiro, Portugal; 4 Department of Biology, University of Konstanz, Konstanz, Germany; 5 Institute of Anatomy, University of Zürich, Zürich, Switzerland; 6 School of Laboratory Medicine, Kwazulu-Natal University, Durban, South Africa; Cajal Institute, Consejo Superior de Investigaciones Científicas, Spain

## Abstract

Organized flight of homing pigeons (*Columba livia*) was previously shown to rely on simple leadership rules between flock mates, yet the stability of this social structuring over time and across different contexts remains unclear. We quantified the repeatability of leadership-based flock structures within a flight and across multiple flights conducted with the same animals. We compared two contexts of flock composition: flocks of birds of the same age and flight experience; and, flocks of birds of different ages and flight experience. All flocks displayed consistent leadership-based structures over time, showing that individuals have stable roles in the navigational decisions of the flock. However, flocks of balanced age and flight experience exhibited reduced leadership stability, indicating that these factors promote flock structuring. Our study empirically demonstrates that leadership and followership are consistent behaviours in homing pigeon flocks, but such consistency is affected by the heterogeneity of individual flight experiences and/or age. Similar evidence from other species suggests leadership as an important mechanism for coordinated motion in small groups of animals with strong social bonds.

## Introduction

Collective motion of wild animals has fascinated generations of scientists, but the social rules governing this phenomenon have only recently begun to be unveiled. This has been facilitated by technological innovations (such as GPS) providing the tools to accurately trace the behaviour of individuals in the social context [1]. More than the coordination itself, the temporal offset of individual behaviours and movement is highly informative regarding decision-making in social groups [2]. When and why do some individuals move earlier than others within their social group? And to what extent do others follow them? These are fundamental questions addressed by current research on animal sociality.

Small groups of social animals routinely have to cope with the need of reaching consensus in order to maintain group cohesion. This can be achieved in a diversity of ways, not necessarily involving all group members in the decision-making process [3]. In leadership, as opposed to participatory democracy, group members rely on the decisions made by one or few leaders in the group. This can be advantageous when leaders possess superior information and speed up the decision-making process, but can also promote unbalanced cost-benefit trade-offs among group members [3], [4]. Leadership has been documented for a substantial number of species, but most studies rely on motion initiation rather than motion guidance behaviour by group members [5]. Yet, the latter context is highly relevant for our understanding of social navigation and migration [6].

Homing pigeons (*Columba livia*) have long been used as model species in animal navigation studies and more recently in pioneering research on social navigation [7]–[9]. Pigeons are known to develop stereotyped homing routes when trained as singletons. These “flight signatures” were used to investigate the social interaction among pairs of birds in joint homing flight [7], [10]–[12]. Leadership was found to emerge when the paired pigeons reached a critical level of route disagreement [7]. A different approach allowing for the study of social interaction in whole flocks of pigeons was proposed by Nagy et al. [13]. This method relied on high resolution GPS tracking in order to find temporal offset in directional changes of different animals in the group. Individuals are recognized as playing a leading role in the flock when flock mates copy their directional choices with a quantifiable time delay. Interestingly, the leader-follower relationships were found to be transitive among multiple pairwise comparisons, showing that the navigational decision-making process in pigeon flocks is hierarchical. This was confirmed by another two studies performed in similar conditions [14], [15]. In particular, Flack et al. [14] have shown that flight hierarchies remain unchanged when additional training is given to a subset of individuals in the flock. While these findings support the idea of an inflexible, leadership-based structure in pigeon flocks, the stability of such structure over time and among different flight contexts deserves further research. For instance, flocks used by Nagy et al. [13], [15] and Flack et al. [14] comprised animals of different ages and flight experience. These factors were recently shown to influence leadership in homing pigeons [10], [16] and could have promoted an over-expression of flock hierarchical structuring in the studies cited above. Moreover, it has been shown that pairs of pigeons with low levels of route conflict take intermediate routes, indicating decision-sharing rather than leadership [7].

In this study we investigated the temporal stability of leadership in small pigeon flocks over two time scales: (1) within the same flight, and (2) between flights (conducted on different days); and among two social contexts: (1) flocks containing pigeons of different ages and flight experience, and (2) flocks where all pigeons were of the same age and had the same flight experience. We used the directional correlation delay method proposed by Nagy et al. [13] in order to find leader-follower relationships between flock mates. Instead of examining the transitive nature of such relationships however, we determined their repeatability over time as an indicator of stability in the flock decision-making structure. Our approach has the advantage of providing a quantifiable output in flock structures ranging from purely egalitarian to inflexible and leadership-based.

## Materials and Methods

### Ethics Statement

The experiments described in this study were conducted according to Swiss regulations on animal welfare and experimentation, license 92/2011 issued by the Zurich Cantonal Veterinary Office. This license was approved by an ethics committee (composed by scientists and animal protection organizations), which is responsible for animal welfare and experimentation licensing for all universities in the canton of Zurich. The license generically approves GPS tracking of homing pigeons, which is considered non-invasive and not imposing stress on the animals (severity level 0 according to Swiss classification). The owner of the pigeons is a co-author in this study (H.-P.L.), the lofts being located on his property. The lofts were approved by the Cantonal Veterinary Office as facility for experimental animals.

### Data collection

This study uses two datasets obtained from tracking flying flocks of pigeons during homing trips. The first comprises the published four homing flights of Nagy et al. [13]. The second includes five homing flights conducted for the purposes of this study with homing pigeons housed in Seuzach, Switzerland (N 47°32′49″, E 8°44′19″). In both studies, groups of 9 to 10 pigeons belonging to the same home loft were transported and released repeatedly on different days from a distance of approximately 15 km. Releases conducted for this study were performed between 14 and 26 June 2012. In both studies, pigeons carried miniaturized GPS data loggers allowing for the reconstruction of their homing trips in detail. Experimental pigeons of Nagy et al. [13] were aged between 1 and 5 years and had previous, unbalanced, homing experience, while ours were juveniles (2 to 3 months old) and received balanced homing flight training for the specific conditions of this experiment. Prior to the five experimental flights, our pigeons were released 16 times at increasing distances to the loft from the same direction as the experimental flights. Birds were released from a 78×40×22 cm training basket by opening a 78×22 cm side door that allowed all pigeons to come out at the same time. Nagy et al. [13] used 5 Hz, 16 g GPS loggers, attached to the subjects with backpack elastic harnesses. We used 4 Hz, 25 g GPS loggers (GiPSy1, Technosmart, Italy) attached by a small Velcro strip glued to clipped feathers on the back of the pigeons. The positional error of our GPS loggers was 1.2±1.1 m (average ± standard deviation, measured for all our GPS loggers by comparing reported positions when stationary to their known geographical position). Comparable errors (1–2 m) were reported by Nagy et al. [13]. Further demonstration that positional GPS errors of such magnitude do not affect the directional correlation delay method is provided by Nagy et al. (Supplementary Methods in [13]). In both studies pigeons were trained with dummy loggers, prior to data collection, to adapt their flying to the logger's extra load.

### Data analysis

GPS data collected for this study included time-stamped longitude and latitude logged with a temporal resolution of 0.25 s. Missing data points were rare (not more than two per track), except for bird M during flight number 7 where ca. 20% of the data points were missing. Still, in this case, missing points where widely distributed along the track and thus we decided to include it in the analysis. Geodetic coordinates were transformed into Cartesian coordinates and linearly interpolated for a 0.2 s temporal resolution to allow for direct comparisons with the dataset of Nagy et al. [13]. The dataset of Nagy et al. [13] was made available with Cartesian coordinates and after the missing points had been interpolated. Prior to analysis, tracks from both datasets were selected excluding birds splitting off from the flock, but maximizing flock size and track length. Accordingly, birds not flying in the flock for more than two thirds of the track length were excluded, and tracks were shortened to include those that split for shorter periods. Splits were always definitive (i.e. splitting birds never returned to the flock). In addition, tracks were selected to correspond to periods of continuous flight with speeds above 40 km/h, thus excluding the beginning and ending flight segments where animals frequently did not fly in formation. Releases were characterized by a strong stochasticity of flight directions and speed. Typically it took the animals a few minutes until they gathered in a flock. Similarly, once they reached the loft area animals started to act independently, which often led to the disaggregation of the flock. Leader-follower relationships between flock mates were determined from the directional correlation delay function described by Nagy et al. [13]. For each pair of birds *i* and *j*, the directional correlation delay function is 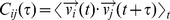, where 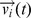 and 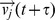 are the normalised velocity vectors at times 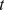 and 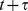 for birds 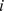 and 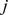, respectively. Velocity vectors were calculated from the geographic coordinates. The maximum value of 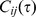 identifies the directional correlation delay time between birds 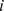 and 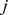. Directional correlation curves were linearly interpolated to a temporal resolution of 0.1 s, which we considered meaningful as time delay for coordinated movement of homing pigeons. Pairs of birds where the maximum value of 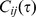 was under 0.9 were considered uncoordinated and were not evaluated for leader-follower relationships. Those cases were typically found when there were birds flying apart from the flock. Directional correlation delay times were simplified into −1, 1 or 0 respectively if the reference individual was the follower (i.e. time delay ≤−0.1 s), the leader (i.e. time delay ≥0.1 s) or had no defined role in the pairwise comparison (i.e. −0.1 s < time delay <0.1 s). These scores are hereafter designated pairwise scores. For graphical purposes, pairwise scores were summed per individual for all pairwise relationships in the flock in order to produce individual leadership ranks. For example, in a flock of 10 birds, a given individual would be assigned with a leadership rank of 9 if it was found to lead in all 9 pairwise comparisons with its flock mates. Leadership consistency was investigated by examining the repeatability of pairwise scores obtained in different flights (between-flight consistency) or in different contiguous segments of the same flight (within-flight consistency). This analysis excluded unresolved pairwise relationships (null pairwise score), and pairs were accounted for in a single direction (if 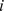 versus 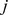 was considered, then 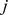 versus 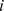 was excluded).

All computations were conducted with the free statistical software R [17]. Repeatability analysis was carried out with the rpt.binomGLMM.multi function from the rptR package [18].

## Results

**Figure 1:**
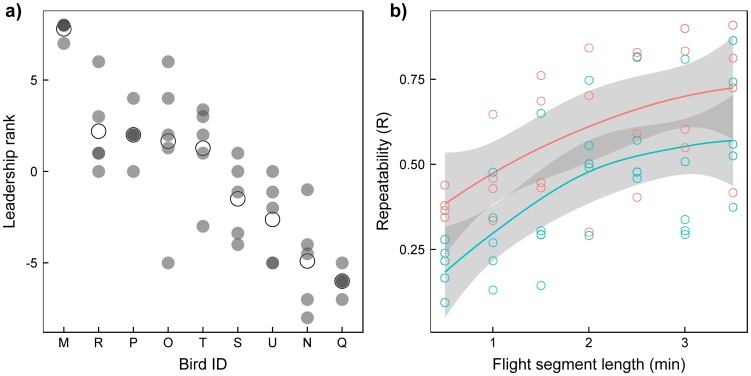
Within-flight leadership consistency. (a) Individual leadership ranks calculated for multiple segments (2.5 min long) of the same flight (illustrated for flight number 8). Average ranks are represented with open dots. The repeatability of leadership pairwise scores (R), P-value and confidence intervals are shown in Table 1. (b) Repeatability of leadership pairwise scores as a function of flight segment length (blue for pigeons with balanced homing experience and red for pigeons with unbalanced homing experience). Dots represent repeatability of different flights when flight segments of different lengths are considered. Trend lines are LOESS curves with 95% confidence intervals.

Overall, individual leadership ranks calculated for multiple contiguous segments in the same flight showed a low dispersion, and pairwise scores showed a high repeatability (see Fig. 1a as an example). A highly significant repeatability was observed for all flights when pairwise scores were calculated from flight segments of 2.5 min (Table 1). Similarly, when computing pairwise scores over segments of different lengths, we found an overall high repeatability (Fig. 1b). However, this repeatability increases with the segment length, meaning that scores calculated from longer segments tend to be more stable over time (Fig. 1b). This increasing trend is asymptotic, suggesting that there is a segment length threshold from which the repeatability of the pairwise scores is maximal (Fig. 1b). Similar trends were found in flights with pigeons of balanced and unbalanced homing experience but a higher repeatability was observed in the first case, independent of the segment length considered. The percentage of undetermined leader-follower relationships was systematically higher in flights with pigeons of balanced homing experience (Table 1).

**Table 1:** Parameters and leadership consistency of flights in the two compared datasets.

Dataset	Flight nr	Flock size	Track length (min.)	Unresolved relationships (%)	R	Confidence interval	P-value
Unbalanced homing experience	1	8 (10)	13	9.4	0.59	[0.24, 0.71]	0.001
	2	7 (9)	15	11.4	0.40	[0.08, 0.57]	0.002
	3	7 (9)	17	21.6	0.82	[0.43, 0.85]	0.001
	4	8 (9)	17	9.5	0.83	[0.54, 0.85]	0.001
Balanced homing experience	5	8 (10)	19	41.3	0.48	[0.12, 0.63]	0.001
	6	8 (10)	17	34.9	0.46	[0.11, 0.62]	0.001
	7	7 (10)	15	41.0	0.57	[0.11, 0.75]	0.001
	8	9 (10)	15	27.3	0.81	[0.49, 0.82]	0.001
	9	9 (10)	16	35.0	0.48	[0.15, 0.61]	0.001

Leadership consistency can be evaluated from the repeatability of pairwise scores (R) computed for multiple contiguous segments (2.5 min long) of the same flight. P-values and confidence intervals for R were generated by randomization (1000 permutations) following Schielzeth and Nakagawa [18]. Unresolved relationships correspond to relationships with null pairwise score. Flock size excludes birds that split from the flock during the homing flight; the number of birds released is presented between brackets.

**Figure 2:**
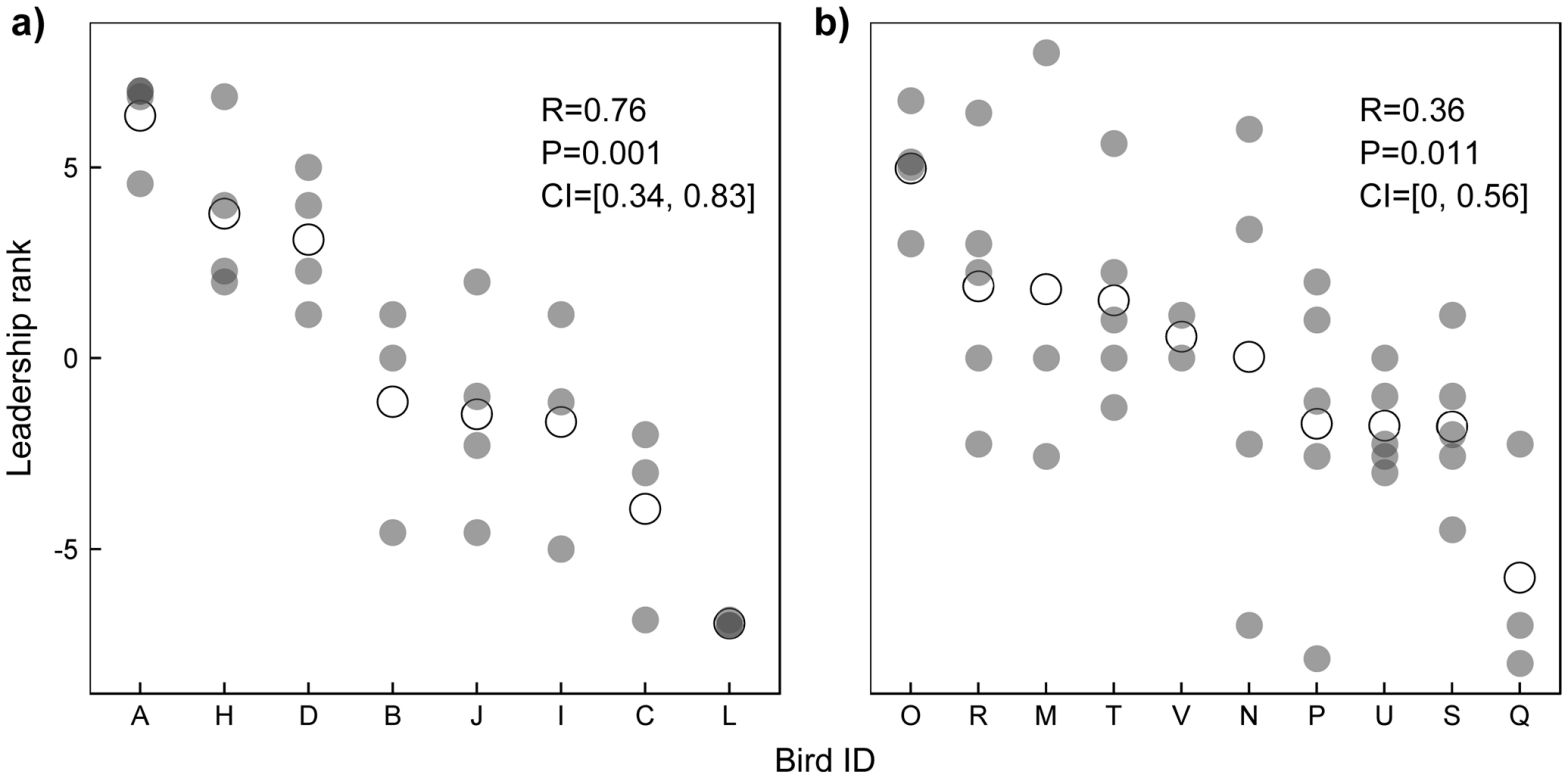
Between-flight leadership consistency. (a) for pigeons with unbalanced homing experience and (b) for pigeons with balanced homing experience. Dots represent individual leadership ranks calculated for multiple flights with the same pigeons. Average ranks per individual are represented by open dots. Leadership consistency can be evaluated from the repeatability of pairwise scores (R). P-values and confidence intervals for R were generated by randomization (1000 permutations) following Schielzeth and Nakagawa [18].

Pairwise scores calculated for multiple flights also showed significant repeatability in both datasets (Fig. 2). However, scores calculated for flights with pigeons of unbalanced homing experience were by far more repeatable. This can be also observed by the higher dispersion of ranks in Fig. 2b when compared with Fig. 2a.

## Discussion

We have shown that patterns of leadership in small flocks of homing pigeons are highly consistent over time and among different social contexts. Pigeons showed significant repeatability in their pairwise leadership scores and low dispersion in leadership ranks both within the same flight and between different flights, meaning that they tend to play stable roles in the navigational flock decisions over time. A significant consistency of leadership was observed in flocks comprising individuals of different age and flight experience, but also in flocks where these factors were balanced. Yet, the degree of repeatability was higher when flocks were diverse in age and flight experience, showing that the robustness of this phenomenon is affected by flock composition. We also have shown that the repeatability of leadership increases with the track length, the increase being asymptotic in both datasets. This can be due to either a higher variance in the coordination of individuals for narrow time frames or caused by the GPS positional error, which is expected to be more influential in small datasets.

These findings are overall in line with those reported by Nagy et al. [13], [15] and Flack et al. [14] showing that navigational decision-making in small pigeons flocks tends to be non-egalitarian and stable over time. However, our results show that balanced homing experience can promote democratic decisions among flock members. In fact, the lower repeatability found in the flocks with balanced homing experience matched a higher number of unresolved leader-follower relationships. These relationships were found invariably between pairs of similar leadership rank in the flock, suggesting decision-sharing between them. These findings are in contrast to those of Flack et al. [14] showing no effect of homing experience on leadership-based flock structures of homing pigeons. However, the two studies varied considerably with regards to the experimental setups adopted to test the same predictions. While our study specifically compares flocks with balanced and unbalanced homing experience, Flack et al. [14] provided additional training to a subset of pigeons flying in flocks with unbalanced homing experience. We believe that the high diversity of homing experience present in the control flocks of Flack et al. [14] could have obscured the manipulations conducted.

The co-existence of leadership and decision-sharing in pigeon flocks has been proposed before [16], [19] and matches the findings of Biro et al. [7] and Freeman et al. [11] who showed a range of decision-making outcomes in homing pigeon pairs. While our results suggest that pigeon flocks may circumstantially include democratic decisions, they are unlikely to be fully egalitarian. This would require a perfect balance in the leading motivation of flock mates, which is rather unlikely in natural circumstances [5]. In fact, conflicts of interest are commonplace among social animals and result inevitably in the emergence of leadership [2], [20]. In our experimental flocks we controlled for the two factors previously shown to influence the leading motivation of homing pigeons, age and flight experience [10], [16], and still we found consistent patterns of leadership. This indicates that leadership is caused by alternative factors (possibly intrinsic such as temperament [21], [22]) but magnified by flight experience and/or age. We must emphasise, however, that experimental flocks were largely composed of the same individuals in both datasets, and thus they should not be interpreted as full replicates. Future studies aiming to understand the effects of flight experience on leadership should include several independent flocks per experimental condition. We also emphasise that our findings do not necessarily apply to larger groups of animals such as fish schools, insect swarms or large flocks of birds. Our experiment was conducted with socially familiar animals moving in small numbers. Thus, besides knowing each other very well, our animals were likely to interact with all their flock mates during each flight. Conditions are certainly different in large animal groups where social interactions are limited to a range of neighbours and typically social bonds are inexistent or ephemeral. Alternative mechanisms have been proposed and should be considered in order to explain the coordinated motion of larger biological aggregates [23], [24].

Our findings fit with the growing evidence of leadership as a mechanism for coordinated motion in small groups animals with strong social bonds [5], although attempts to generalize these findings should be conducted under the consideration that leadership has been quantified in a diversity of ways that are not necessarily comparable. Although leadership may be a widespread phenomenon among social animals, our study is among the few that evaluated its consistency (see also [25], [26]). Lack of consistency (temporal or contextual) between leadership events dramatically constrains their biological significance. Consistent leadership, on the other hand, reflects heterogeneity of individual social traits, which can be affected by natural selection and determine evolutionary pathways [5]. With an increasing number of bio-logging techniques becoming available, scientists now have the opportunity to survey animal social interactions over time and varying contexts with great precision. This provides an ideal framework to investigate the true nature of animal societies.
